# Are we conSTRucting the best treatment regimens for all patients with HIV infection?

**DOI:** 10.7448/IAS.15.6.18095

**Published:** 2012-11-11

**Authors:** D Friday, J Evans-Jones, N Steedman

**Affiliations:** 1 Countess of Chester Hospital NHS Foundation Trust, Department of Sexual Health, Chester, UK; 2 Western General Hospital, Department of Infectious Diseases, Edinburgh, UK

## Abstract

The British HIV Association (BHIVA) 2012 guidelines for the treatment of HIV recommend patients start combination antiretroviral therapy (ART) containing tenofovir and emtricitabine as the nucleos(t)ide reverse transcriptase inhibitor (NRTI) backbone. BHIVA third agent preferred choices comprise efavirenz, boosted atazanavir, boosted darunavir and raltegravir, and the guidelines further state that ‘fixed dose combinations (FDC) of drugs can increase adherence’ [1]. Atripla is currently the only available single-tablet regimen (STR) for the treatment of HIV which contains a combination of BHIVA preferred first-line antiretrovirals (tenofovir, emtricitabine and efavirenz). In addition, studies have shown that Atripla can improve adherence, treatment satisfaction and outcomes for patients infected with HIV [2]. A retrospective case note review was conducted for all HIV-positive patients attending a UK HIV centre and receiving ART. The purpose was to ascertain the proportion of patients receiving BHIVA preferred ART in its simplest dosing format (in this case Atripla) and to investigate whether there were clinically or virologically appropriate reasons why patients not on Atripla were prescribed more complex drug regimens. The total number of patients receiving ART at the time of review was 142, of which 47 (33%) were currently taking Atripla. Of the remaining 95 patients, 30 (32%) had taken Atripla or some of its components in the past and been changed from this for valid clinical or virological reasons. In addition, there were a further 34 cases (36%) where Atripla had never been offered to the patient for appropriate reasons and documentation of 8 instances in which Atripla had been declined by the patient. There remained, however, 28 cases (29%) where there was no documentation of Atripla having been considered or offered, and no apparent contraindications to the STR or its components. This included 4 patients with an elevated cardiovascular risk, all of whom were taking an abacavir-containing ART regimen at the time of review. Despite extensive professional guidance on preferred ART regimens and evidence to suggest that STR can increase adherence and patient satisfaction there remain patients in clinical care taking more complex drug regimens with no clear indication for this. We would encourage physicians to identify such patients and to discuss with them their treatment options in the light of advances in ART combination preparations.
